# Analysis of out-of-hospital cardiac arrest in Croatia – survival, bystander cardiopulmonary resuscitation, and impact of physician’s experience on cardiac arrest management: a single center observational study

**DOI:** 10.3325/cmj.2016.57.591

**Published:** 2016-12

**Authors:** Anita Lukić, Ileana Lulić, Dinka Lulić, Zoran Ognjanović, Davorin Cerovečki, Siniša Telebar, Ivica Mašić

**Affiliations:** 1 Department of Anesthesiology, Reanimatology and Intensive Care Medicine, Varaždin General Hospital, Varaždin, Croatia; 2 Institute of Emergency Medicine Varaždin, Varaždin, Croatia; 3 Department of Physiology, Nursing studies, Technical College in Bjelovar, Bjelovar, Croatia; 4 Department of Anesthesiology, Reanimatology and Intensive Care Medicine, Clinical Hospital Centre Zagreb, Zagreb, Croatia; 5 Department of Emergency Medicine, Clinical Hospital Centre Zagreb, Zagreb, Croatia

## Abstract

**Aim:**

To analyze the initial rhythm, bystander cardiopulmonary resuscitation (CPR) rate, and survival after out-of-hospital cardiac arrests (OHCA) in Varaždin County (Croatia), and to investigate whether physician’s inexperience in emergency medical services (EMS) has an impact on resuscitation management.

**Methods:**

We reviewed clinical records and Revised Utstein cardiac arrest forms of all out-of-hospital resuscitations performed by EMS Varaždin (EMSVz), Croatia, from 2007-2013. To analyze the impact of physician’s inexperience in EMS (<1 year in EMS) on resuscitation management, we assessed physician’s turnover in EMSVz, as well as OHCA survival, airway management, and adherence to resuscitation guidelines in regard to physician’s EMS experience.

**Results:**

Of 276 patients (median age 68 years, interquartile range [IQR] 16; 198 male; 37% ventricular fibrillation/ventricular tachycardia, bystander CPR rate 25%), 80 were transferred to hospital and 39 were discharged (median survival after discharge 23 months, IQR 46 months). During the 7-year study period, 29 newly graduated physicians inexperienced in EMS started to work in EMSVz (performing 77 resuscitations), while 48% of them stayed for less than one year. Airway management depended on physician’s EMS experience (*P* = 0.018): inexperienced physicians performed bag-valve-mask ventilation (BMV) more than the experienced, with no impact on survival rate. Physician’s EMS experience did not influence adherence to resuscitation guidelines (*P* = 0.668), survival to hospital discharge (*P* = 0.791), or survival time (*P* = 0.405).

**Conclusion:**

OHCA survival rate of EMSVz resuscitations was higher than in Europe, but bystander CPR needs to be improved. Compared to experienced physicians, inexperienced physicians preferred BMV over intubation, but with similar adherence to resuscitation guidelines and survival after OHCA.

Every year, roughly 350 000-700 000 Europeans experience out-of-hospital cardiac arrest (OHCA) (1), but only 9% of them will survive to hospital discharge (1). It is well known that the outcome of OHCA depends on a number of patient’s intrinsic factors, but the critical determinants of survival are immediate bystander cardiopulmonary resuscitation (CPR) and early defibrillation, as it is repeatedly pointed out by the European Resuscitation Council (ERC) (2). Although the incidence, survival rate, and bystander CPR rate is known in many countries (1), there are still no data about the features of OHCA in Croatia (3), which could provide a basis for the improvement of OHCA management and survival.

Another factor that improves survival after OHCA is the presence of a physician in the emergency medicine service (EMS) team (4,5). Unfortunately, high fluctuation and turnover of physicians in EMS have been noticed, with a constant in-flow of newly graduated and inexperienced doctors. Previous studies showed lower success rates and higher complication rates when endotracheal intubation is performed by inexperienced prehospital emergency medicine staff (6-12), which triggered the change of ERC guidelines. Therefore, ERC 2010 Guidelines recommended that tracheal intubation “should be used only when trained personnel are available to carry out the procedure with a high level of skill and confidence” (13,14). On the other hand, despite clear guidelines, there are no studies investigating the impact of physician’s experience in EMS on the actual airway management style and the adherence to the resuscitation guidelines in prehospital setting or outcomes after OHCA.

Therefore, the aim of this study was to investigate the features of OHCA: the initial rhythm, bystander CPR rate, and survival after OHCA in Varaždin County (Croatia). Also, we investigated the turnover rate of physicians in prehospital EMS, and whether physician’s experience in EMS had an impact on the actual airway management, OHCA survival rate, and the adherence to resuscitation guidelines.

## Methods

### Study design and data sources

In this observational study we reviewed the data from out-of-hospital resuscitations collected by Revised Utstein cardiac arrest data collection forms (15). The forms provided the following data: patient age and sex, cause and location of cardiac arrest (out-of-/in-hospital), presence of witnesses to the arrest (and time of arrest, if witnessed), bystanders’ treatment before EMS arrival (bystander CPR and defibrillation witnessed by EMS team or reported to EMS team), time of collapse, call receipt, vehicle stop, and first rhythm analysis, as well as the time when EMS started the resuscitation, initial rhythm, data on defibrillations, ventilation and drugs, and the return of spontaneous circulation (ROSC).

### Setting, participants, and study size

The study was conducted at the Institute of Emergency Medicine Varaždin, in Varaždin, Croatia – Emergency Medical Service Varaždin (EMSVz). The EMSVz covers an area of 346 km^2^, populated by 92 755 inhabitants. During the study period, EMSVz had ten Advance Life Support teams, each with a fully equipped road vehicle. Every team consisted of three members: a physician, registered nurse, and professional driver (of non-medical education).

We analyzed the data on all out-of-hospital resuscitations OHCA performed by EMSVz from January 1, 2007 until December 31, 2013. Although we included all resuscitations performed in this time frame, with no exclusions, it should be emphasized that, according to guidelines (16-18), EMSVz does not perform resuscitation in the cases were resuscitation is considered futile (such as the presence of rigor mortis, dependent lividity, decapitation, or decomposition). These cardiac arrests were not considered for this study – just as they are not considered for other studies either.

After the resuscitation, the patients were brought to the local secondary care hospital, Varaždin General Hospital. There were no cases of cardiac arrest of traumatic origin. This study is approved by the Ethics Committee of the Institute of Emergency Medicine Varaždin.

### Outcomes

We analyzed patients’ age (in years), EMSVz response time (from call to EMSVz CPR, in minutes), and survival rate. The survival rate was assessed using two metrics: a) the number of patients discharged from the hospital divided by the total number of resuscitation attempts (total survival); b) the number of patients discharged from the hospital divided by the number of patients brought to the hospital. Survival time after hospital discharge was analyzed as the time from cardiac arrest to death if discharged patient died, or until April 2014 when the follow up was discontinued due to the end of the study.

In addition, resuscitations were analyzed in regard to physician’s experience in EMS. Inexperienced physicians were considered those working in EMS for less than one year, while experienced physicians were considered those working in EMS for more than one year. Before starting any field-work, a newly hired physician had to attend in-house courses along the lines of European Resuscitation Council courses (19), covering trauma management and advanced adult and pediatric life support, and including different manners of airway management. After completing these courses, all new physicians started working under the supervision of a mentor for at least two months before getting the approval to work unsupervised.

Next, we analyzed the adherence to the ERC Guidelines in regard to physician’s experience in EMS. After processing Utstein forms (15), cardiac arrest management was compared to the ERC Guidelines and algorithms for advanced life support. The deviations from these algorithms were recorded as non-adherence to the guidelines. The valid guidelines were considered: ERC 2005 Guidelines (13), for the study period from January 1, 2007 till October 17, 2010 and ERC 2010 Guidelines (14) for the study period from October 18, 2010 (when the 2010 Guidelines were published) to December 31, 2013, when the data acquisition ended. Since the medical personnel in EMSVz were forewarned and repeatedly reminded about the release of new ERC 2010 Guidelines, they were expected to implement the new Guidelines promptly. Therefore, all resuscitation attempts from October 18, 2010 were planned to be analyzed in comparison to ERC 2010 Guidelines. The first resuscitation attempt after the publishing of ERC 2010 Guidelines was on October 23, 2010.

### Statistical analysis

Data on patients’ age, sex, survival rate, survival time, and adherence to the valid guidelines were summarized using descriptive statistics based on the normality of distribution (D'Agostino-Pearson test for normal distribution). Categorical variables were compared using Fisher exact test with Bonferroni’s correction for multiple testing when needed, while continuous variables were compared using the Mann-Whitney test for independent samples. Statistical difference in survival time was assessed by Logrank test. All statistical analyses were performed using MedCalc 9.5.1.0 (MedCalc Software, Mariakerke, Belgium). *P* values lower than 0.05 were considered statistical significant, if not indicated otherwise (ie, Bonferroni’s correction). This manuscript is organized following STROBE statement for cohort studies: Strengthening the Reporting of Observational Studies in Epidemiology (20).

## Results

### Participants

**Figure 1:**
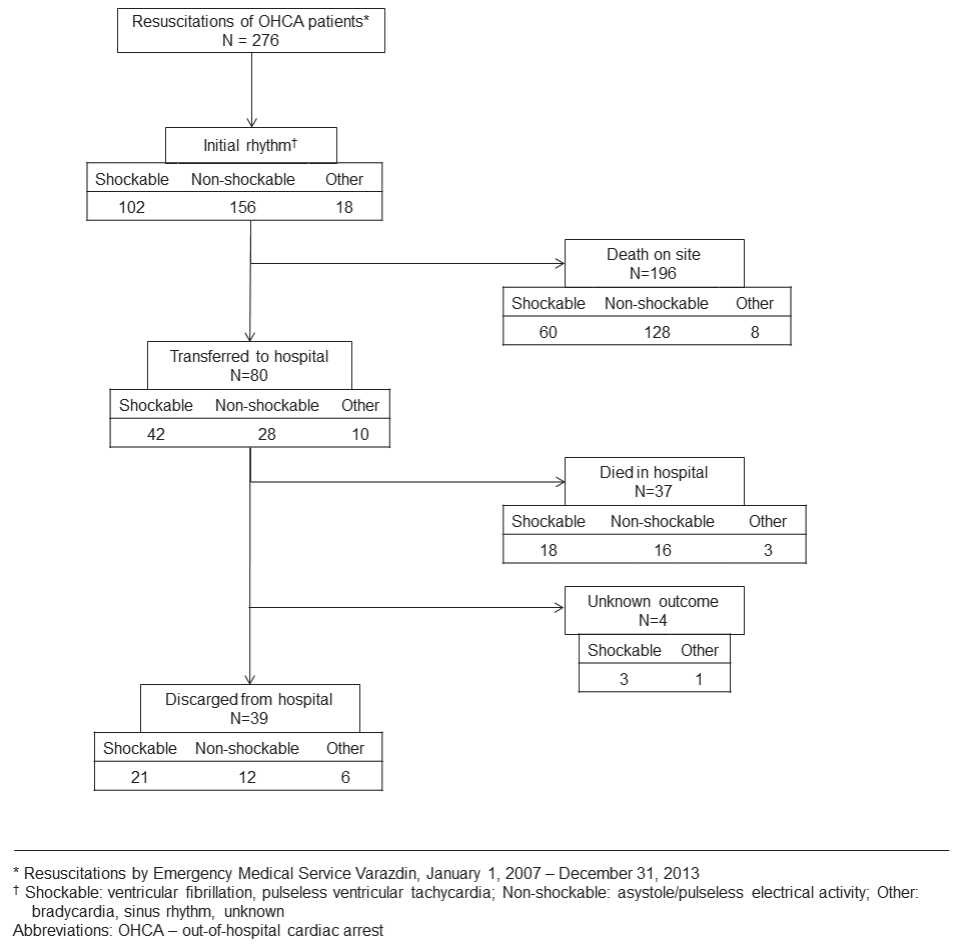
Patients flow-chart in the observational study on features of out-of-hospital cardiac arrest and impact of prehospital physician’s experience on resuscitation management. The study was performed from 2007 to 2013 by Emergency Medical Service Varaždin.

During the 7-year study period, EMSVz performed 276 out-of-hospital resuscitations on 274 patients (two patients were resuscitated twice). Flow diagram of the patients is shown in Figure 1.

### Descriptive data

The median age of 198 male and 78 female patients with OHCA was 68 years (interquartile range [IQR] 16; range 0-92 years), with no difference between the sexes (*P* = 0.183). Median EMSVz response time was 6 minutes (IQR 6; range 0-34 minutes). Data about bystanders CPR were recorded in 159/276 patients – bystanders started CPR in 39/159 (25%) cases.

Initial rhythm was recorded as follows: 102 patients (37%) had shockable rhythm, 156 (57%) had non-shockable rhythm, and 18 patients had some other initial rhythm (sinus rhythm, bradycardia, or unknown) (Table 1). Patients with shockable and non-shockable rhythms were resuscitated equally by inexperienced (No. shockable vs non-shockable: 31 vs 40) and experienced physicians (No. shockable vs non-shockable: 71 vs 116), *P* = 0.397. Resuscitations continued for a median of 29 minutes (IQR 16 minutes, range 2-88 minutes), and resuscitations of ventricular fibrillation were significantly longer (median 30 minutes, IQR 22 minutes, range 7-88 minutes) than resuscitations of patients with non-shockable rhythms (median 26 minutes, IQR 12 minutes, range 2-75 minutes), *P* = 0.015.

**Table 1:** Features of out-of-hospital cardiac arrest in the observational study of 276 resuscitations performed from 2007 to 2013 by Emergency Medical Service (EMS) Varaždin

Variable	Died at site	Transferred to hospital	P*	Died in hospital	Discharged from hospital	Unknown hospital outcome	*P*^†^
Response time (call to cardiopulmonary resuscitation, minutes)^§^	7 (0-32, 6)	5 (0-34, 5)	0.020^‡^	6 (0-18, 6)	5 (1-34, 5)	-	0.842^‡^
Duration of resuscitations (minutes)^§^	30 (4-88, 16)	25 (2-70, 18)	0.150^‡^	32 (13-70, 17)	20 (2-60, 14)	-	<0.001^‡^
Patients’ age (years)^§^	68 (0-92, 14)	66 (13-89, 17)	0.064^‡^	67 (13-87, 22)	67 (27-84, 16)	-	0.928^‡^
Sex (No. of patients)							
male (198)	145	53	0.238^¶^	22	30	1	0.083^¶^
female (78)	51	27		16	8	3	
Initial rhythm (No. of patients)^ǁ^							
shockable (102)	60	42	<0.001^¶^	18	21	3	0.460^¶^
non-shockable (156)	128	28		16	12	0	
other (18)	8	10	-	3	6	1	-
Physician’s experience in EMS (No. of resuscitated patients)							
<1 year (78)	58	20	0.656^¶^	10	8	2	0.791^¶^
>1 year (198)	140	58		28	27	3	

*Dead at the site vs transferred to hospital.

†Died in hospital vs discharged from hospital.

‡Mann-Whitney test for independent samples.

§Median (range, IQR).

¶Fisher exact test.

ǁShockable: ventricular fibrillation, pulseless ventricular tachycardia; non-shockable: asystole/pulseless electrical activity.

### Survival

Out of 276 out-of-hospital resuscitation attempts, 39 (14%) patients lived until the hospital discharge (21/102 of patients with initial shockable rhythm, 12/156 with initial non-shockable rhythm, and 6/18 with other initial rhythms [discharged patients with shockable vs discharged patients with non-shockable rhythm: *P* = 0.004]). Survival until hospital discharge was associated to the duration of out-of-hospital CPR, where patients who sustained shorter out-of-hospital CPR to ROSC were more likely to be discharged from the hospital than patients who experienced longer out-of-hospital CPR (*P* < 0.001). However, survival until discharge was associated with none of the other analyzed variables (Table 1).

Median survival time after hospital discharge was 23 months (IQR 46, range 0-68 months), and it was weakly associated with the patient’s age (*P* = 0.049). However, it was not associated with patients’ sex (*P* = 0.728), initial rhythm (*P* = 0.851), or the physician’s experience in EMS (*P* = 0.405) (Table 2). In addition, there was no correlation of survival time of discharged patients and EMS response time (*P* = 0.176) or duration of out-of-hospital CPR (*P* = 0.201). The 30-day survival rate after the discharge was 32/276 (12%), 90-day survival rate was 28/276 (10%), while one-year survival was 23/276 (8%).

**Table 2:** Survival time after hospital discharge following out-of-hospital cardiac arrest in the observational study of 276 resuscitations performed from 2007 to 2013 by Emergency Medical Service (EMS) Varaždin

Variable	Survival time of patients			
	all*		discharged^†^	
	months^§^	*P*^¶^	months^§^	*P*^¶^
Sex				
male	0 (0-68, 0)	0.053	31 (0-68, 50)	0.728
female	0 (0-64, 0)		22 (0-68, 28)	
Initial rhythm^‡^				
shockable	0 (0-66, 0)	0.005	31 (0-68, 44)	0.851
non-shockable	0 (0-68, 0)		24 (0-68, 60)	
Physician’s experience in EMS				
<1 year	0 (0-57, 0)	0.785	28 (0-68, 20)	0.405
>1 year	0 (0-68, 0)		20 (0-68, 52)	

*All patients with the attempt of resuscitation.

**†**Patients discharged from hospital.

‡Shockable: ventricular fibrillation, pulseless ventricular tachycardia; non-shockable: asystole/pulseless electrical activity.

§Median (range, IQR).

¶Logrank test.

### Physicians’ turnover rate and impact of physician’s experience on resuscitation features

At the start of the study, there were five physicians with 6 to 25 years of experience in EMS, and all of them continued to work in EMSVz for the duration of the study. During the 7-year study period, 29 physicians with no experience in EMS started working at EMSVz, just after graduating from medical school. One physician stayed in EMSVz for 4 years, three stayed for 3-4 years, six for 2-3 years, five for 1-2 years, and fourteen for less than one year (ten of them only for 3-4 months). Experienced physicians performed 197 (72%) resuscitations, while inexperienced physicians performed 77 (28%) resuscitations.

### Airway management and the adherence to the ERC Guidelines

Airway management depended on the physician’s experience in EMS (inexperienced vs experienced *P* = 0.018) (Table 3). Inexperienced physicians performed bag-valve-mask ventilation (BMV) using Guedel airway more frequently than experienced physicians (21% vs 9%, *P* = 0.012). Besides successful intubation attempts, attempts to intubate were also recorded, but only in 157 resuscitations during the period from 2010 to 2013 (Table 4). Inexperienced physicians made significantly fewer intubation attempts than experienced physicians (26% vs 60%, *P* < 0.001), and when they tried to intubate they were less successful than their experienced colleagues (67% vs 95%, *P* = 0.007). There were no attempts at rapid sequence intubation. The applied method of airway management was not associated with overall survival rate to discharge from the hospital after out-of-hospital CPR (*P* = 0.805).

**Table 3:** The impact of physician’s experience in emergency medical service on airway management in 276 resuscitations performed from 2007 to 2013 by Emergency Medical Service Varaždin

Airway management	Experience, No (%)*		*P^†^*
	inexperienced	experienced	
Guedel airway + bag-valve-mask ventilation	16 (21)	17 (9)	0.012
Endotracheal intubation	18 (23)	89 (45)	<0.001
Supraglottic device	43 (55)	88 (44)	0.181
Other/unknown	1 (1)	4 (2)	1.000
Total	78 (28)	198 (72)	0.018

*Working as emergency medicine field physician: inexperienced <1 year; experienced >1 year.

**†**Fisher exact test, Bonferroni's correction for multiple testing: significant when *P* < 0.017.

**Table 4:** The impact of physician’s experience in emergency medical service on endotracheal intubation success recorded for 157 of 276 resuscitations performed from 2010 to 2013 by Emergency Medical Service Varaždin. In 119 Utstein forms there was no record about unsuccessful intubation attempts

Endotracheal intubation	Experience, No (%)*		*P^†^*
	inexperienced	experienced	
No attempt	43 (74)	40 (40)	<0.001
Attempts	15 (26)	59 (60)	
successful	10 (67)	56 (95)	0.007
unsuccessful	5 (33)	3 (5)	
Total	58	99	

*Working as emergency medicine field physician: inexperienced <1 year; experienced >1 year.

**†**Fisher exact test.

Physician’s experience in prehospital EMS was not associated with survival rate until hospital discharge (*P* = 0.791, Table 1), or median survival time after hospital discharge (*P* = 0.405, Table 2). We found no differences in the adherence to the ERC Guidelines between inexperienced and experienced prehospital EMS physicians (*P* = 0.668).

## Discussion

This is the first ever study analyzing the features, bystander CPR rate, and survival after OHCA in Croatia, and we found survival rate after OHCA in Varaždin County to be higher than overall survival rate in Europe, with bystander CPR started in one quarter of resuscitations.

This is also the first study investigating the turnover rate of inexperienced physicians in prehospital emergency medical service, with the impact of the physician’s inexperience on OHCA survival rate, and the physician’s adherence to resuscitation guidelines. Although we showed a high turnover rate of inexperienced physicians in our prehospital emergency department, their inexperience did not influence their adherence to the resuscitation guidelines or patients’ survival after OHCA. In addition, while other studies have investigated endotracheal intubation failure and success rates in regard to physician’s experience, this study investigated the actual airway management style of inexperienced physicians in prehospital setting, and we showed different approach to airway management in regard to physicians’ experience.

### Survival after OHCA in Varaždin County

Today, despite the development of CPR and electrical defibrillation as treatment modalities more than half a century ago, OHCA still presents one of the major public health problems, with survival rates remaining relatively low worldwide: in Europe 9%, North America 6%, Asia 3%, Australia 13% (1). While numerous studies investigated survival rates after OHCA in different European countries, this is the first study investigating different features of OHCA resuscitation in Croatia, including the survival rate (3). Although our results are far from the survival rate of 43% in Netherlands (21) or 35% in Osaka Prefecture, Japan (22), the survival rate until discharge after OHCA of 14% in Varaždin County exceeds OHCA overall survival rate in Europe (9%) (1), part of Poland (10%) (23), or Czechia (9%) (24), with the latter two having similar cultural characteristics as Croatia. Such a good survival rate is probably due to the prompt response time of 6 minutes and physician-staffed EMS team, but we also showed that the survival rate to discharge was associated with the conduction of bystander CPR. Although we had data on bystander CPR for only 58% of the analyzed OHCA, bystanders started CPR in 25% of them, which undoubtedly led to better outcomes of these patients. This bystander CPR rate is slightly higher than in Germany (19%) (25) or Slovenia (22%) (26). Despite of this, we feel that our goal should be set higher, toward the bystander CPR rates of about 40%, as is the case in the United States (27) and England (28), or 76% in the Netherlands (21).

Hence, although both survival rate and bystander CPR rate found in this study are quite good, there is plenty of room for improvement. As EMS field physicians and medical technicians, we believe that the first step would be the development of a national OHCA registry to establish OHCA incidence and outcomes at national and local levels. Using the registry we could identify both deficiencies and strengths, which is the key first step in the improvement of OHCA management and progress monitoring. Also, it would be valuable if the registry contained the data pertaining to the frequency and manners of CPR quality measurements, and CPR quality itself; this would provide a basis for the monitoring of CPR quality improvement.

Since almost half of OHCA events are witnessed, but only few witnesses start CPR (29), another essential contribution to the increase in OHCA survival rate would be to improve the bystander CPR rate. As bystander CPR rate could double OHCA survival (30), we need to raise the public awareness that cardiac arrests are not rare events and teach laymen to recognize cardiac arrest, as well as encourage them to start CPR. In our opinion, this could be achieved by: 1) the promotion of basic life support courses; 2) media campaigns showing the dramatic impact of bystander CPR on survival; 3) the implementation of CPR-automated external defibrillators (AED) training in high school curriculums; 4) and better availability of and accessibility to AEDs. Until recently, there were not many AEDs in Croatia. However, as a part of the national program “Start the Heart – Save a Life“ introduced by the Croatian Ministry of Health in 2013 (31), numerous basic life support courses should be delivered to lay persons, and about 200 AEDs should be installed in public places in Croatia. Currently, in Croatia there are 271 available AEDs (32). This may not seem much on the national level, but it must be taken into account that Croatia is a small country, with only 4.2 million inhabitants. Through the implementation of these methods, we hope that bystander CPR rate will increase in the following years, and that it will lead to the improvement of survival rates following OHCA.

In addition to bystander CPR rate, better survival to discharge in our study was associated with shorter duration of out-of-hospital CPR. A possible explanation for better survival of these patients would be that they needed “shorter” CPR only because they were “less ill.” The underlying pathophysiological mechanism can only be speculated about. Bearing in mind that several other studies also reported a link between shorter CPR and better outcome (33-35), but did not focus on the causality, the follow-up studies are called for.

### Physicians’ turnover rate

This study revealed a high physician turnover rate in EMSVz. Almost half of 29 inexperienced physicians who started to work in EMSVz during the 7-year study period stayed for less than a year, with one third of them staying for only a few months. Furthermore, such a turnover rate of newly-graduated physicians in EMSVz, which has just ten teams, must raise concerns, especially since we believe (based on personal communication) that EMSs in other parts of Croatia struggle with the similar fluctuation of physicians. This kind of turnover could be caused by the fact that EMSs have been understaffed, since there was no residency in emergency medicine for out of hospital emergency medical service in Croatia until November 2013. Being understaffed, EMSs have been forced to hire inexperienced physicians. In addition, a huge percentage of these physicians have perceived EMS as a “waiting room” for their desired residency outside of emergency medicine. We believe this happened here, since out of 29 physicians who started to work in EMSVz during the study period, only 2 were interested in residency in emergency medicine (personal communication). Although physician’s inexperience did not influence the adherence to the ERC guidelines or survival rate in this study, we believe this kind of practice is not favorable and it must be changed. A major step in this direction will be the residency in emergency medicine for pre-hospital emergency services, which started in November 2013.

### Association of physician’s experience and airway management

In this study, we recorded differences in airway management in regard to physician’s experience in EMS. Experienced physicians did more endotracheal intubations, while inexperienced physicians did more BMVs with Guedel’s airway. Considering their short training in airway management and the obvious lack of experience that would make them skilled in advanced airway management techniques, endotracheal intubation in particular, we find the preference of our inexperienced colleagues to BMV instead of intubation reasonable, especially since a third of their intubation attempts were unsuccessful. Intubation failure rate in inexperienced physicians is much lower than the 50% found in other prehospital systems also with a low patient volume and providers who did not perform intubation frequently (8,11). However, it is far greater than 5% failure rate in their experienced colleagues found in Sayre’s study (8) and a meta-analysis of intubations success rates of EMS providers (12). Therefore, we agree that endotracheal intubation should be attempted only by well trained personnel who can carry out the procedure with a high level of skill and confidence (2,14,36).

### Physician’s experience and resuscitation features

Inexperienced physicians performed less than a third of all resuscitation attempts, but with similar adherence to the resuscitation guidelines. Similar survival rate and survival time of patients resuscitated by experienced and inexperienced physicians challenge the common belief that “it is better to be treated by an old doctor.”

### Limitations

There are a few limitations of our study. First, we analyzed OHCA survival from a single center with a small number of OHCA events. Because the investigated area covered by EMSVz is more densely populated than the whole state of Croatia, our results might not be generalizable to Croatia as a whole. We would like to emphasize that the studies investigating survival after OHCA in just one part of a country are quite frequent (1), since the establishment of a national registry is a major and demanding project, which is proven by the fact that there are only five national registries of OHCA in the world (37,38). Despite this limitation, this study is important encouragement to the development of a national registry. Second, this study is retrospective and observational in nature, so we were limited only to the data previously recorded on Utstein forms and medical records. If the study design was prospective, we could have asked physicians to fill out the forms and records more completely or collect additional data. Third, we used the Utstein report and medical record. It is possible that using video recordings of OHCA management would provide us more data on differences in OHCA management in regard to physicians’ experience in EMS. Finally, we did not analyze the quality of CPR or timing and organization of resuscitation attempt of inexperienced physicians, only the adherence to the resuscitation algorithms.

In conclusion, our results demonstrate that the OHCA survival rate until hospital discharge in Varaždin County exceeds the overall survival rate in Europe, with the bystander CPR rate matching that in the neighboring countries. Although there was a high turnover rate of newly graduated inexperienced physicians, they followed the ERC Guidelines similarly and with comparable patient survival rates as their more experienced colleagues. On the other hand, inexperienced physicians managed airway differently, being more comfortable with BMV than with endotracheal intubation, as it was recommended in ERC resuscitation guidelines.
